# A Machine learning pipeline to investigate tissue ingrowth in cerebral aneurysms using preclinical animal models

**DOI:** 10.1038/s41598-026-43798-w

**Published:** 2026-03-13

**Authors:** Fatemeh Afsari, Ishaq Ansari, Melanie E. Martinez, Lillian Atchison, Sayat Mimar, Koji Hosaka, Brian Hoh, Pinaki Sarder

**Affiliations:** 1 Division of Nephrology, Hypertension, and Renal Transplantation—Quantitative Health Section, Department of Medicine, College of Medicine, University of Florida, Gainesville, FL 32611 USA; 2 School of Natural Sciences, Caldwell University, Caldwell, NJ 07006 USA; 3 Department of Neurosurgery, College of Medicine, University of Florida, Gainesville, FL 32611 USA

**Keywords:** Cerebral aneurysm, Tissue ingrowth, Histological image analysis, Unet++, Deep learning, Segmentation, Computational biology and bioinformatics, Engineering, Mathematics and computing, Medical research

## Abstract

Cerebral aneurysm is a life-threatening condition characterized by the formation of a saccular bulge in brain blood vessels, which can rupture and lead to severe complications. One treatment involves inserting a soft, flexible wire (coil) into the aneurysm to promote clotting and sealing. Mediators are often used to simulate tissue ingrowth within the sac to stabilize healing and prevent recurrence. However, quantitative assessment of tissue ingrowth in preclinical models remains labor-intensive, subjective, and poorly standardized, limiting the ability to compare therapeutic strategies and healing mechanisms. We developed a robust machine learning (ML) pipeline based on a Unet + + convolutional neural network (CNN), optimized for segmenting and quantifying tissue ingrowth in a preclinical carotid aneurysm mouse model. The model was trained and validated on 64 high-resolution histological images using 10-fold cross-validation. Image preprocessing included resizing, normalization, and augmentation, while post-processing applied thresholding techniques to CNN-generated heatmaps. Our method achieved Dice coefficients of 94.58% for sac segmentation and 95.23% for tissue ingrowth detection, with AUCs of 99.24% and 96.78%, respectively. The model’s predictions showed strong agreement with ground truth ($R^{2}=0.94$), supporting its potential for assessing biological stability and informing clinical decisions. In a blinded evaluation against expert annotations, our AI model achieved the highest agreement (Cohen’s κ) among all raters, demonstrating its potential to provide consistent and expert-level tissue ingrowth assessments. A user-friendly graphical interface was developed, to enable non-technical users to perform segmentation and quantify tissue ingrowth. By providing objective, reproducible metrics of intra-aneurysmal healing, this approach supports mechanistic studies of therapeutic efficacy in preclinical aneurysm models and establishes a foundation for standardized evaluation of pro-healing interventions.

**Supplementary Information:**

The online version contains supplementary material available at 10.1038/s41598-026-43798-w.

## Introduction

**Fig. 1:**
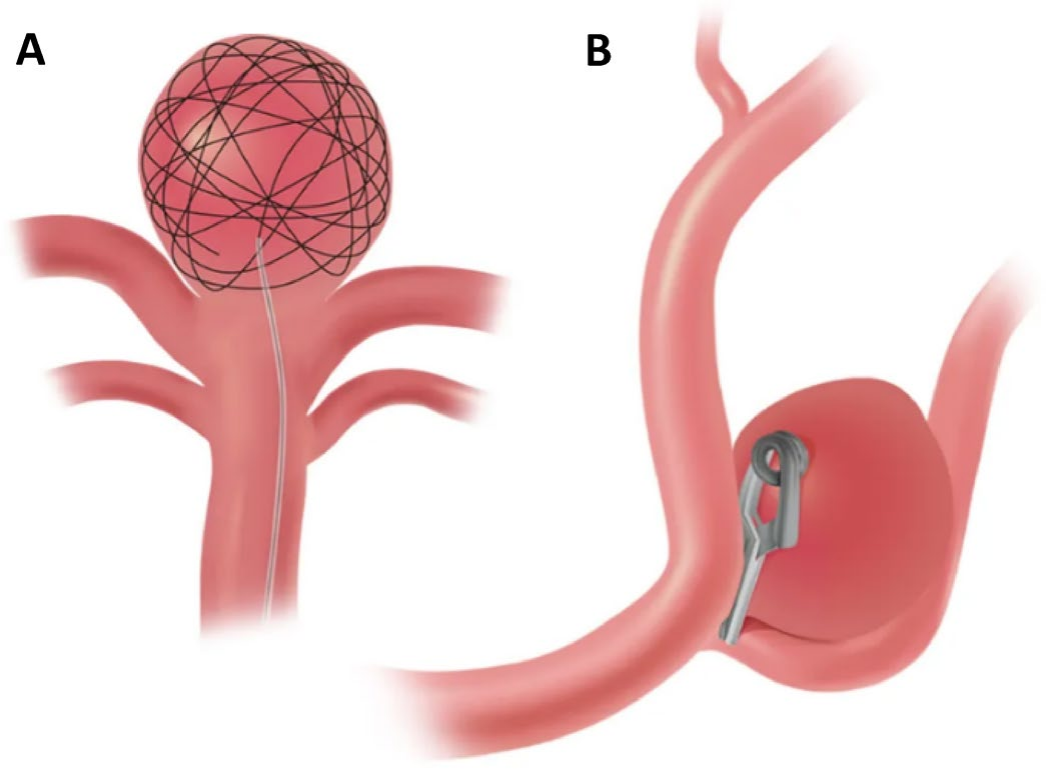
**Comparison of treatment methods for cerebral aneurysms**: (**A**) minimally invasive endovascular coiling, in which coils are deployed within the aneurysm sac to promote thrombus formation and subsequent tissue ingrowth, and (**B**) microsurgical aneurysm clipping via open surgery, which excludes the aneurysm from circulation by placement of a clip at its neck. Adapted with permission from *Brain Aneurysm: Treatments and Illustrations*, UT Southwestern Medical Center, Peter O’Donnell Jr. Brain Institute^8^, under a CC BY 4.0 license. This figure has been modified from the original by the removal of the flow redirection treatment method.

Cerebral aneurysm is a serious and potentially fatal vascular disorder characterized by weakening and ballooning of blood vessel walls in the brain^1,2^. When an aneurysm ruptures, it can cause hemorrhage and stroke, often leading to severe neurological damage or death. Aneurysm clipping, an open surgical procedure, is a treatment for brain aneurysms in which an opening is made in the skull and a small metal clip is placed at the base of the aneurysm to prevent blood flow into it^3^.This method involves the implantation of a graft acquired through surgery, which has direct contact with the blood vessels. Another technique employs endovascular coiling; without the need for open surgery, a catheter is inserted through the arteries of the body and reaches an aneurysm^4^. Very small, soft, and flexible metal coils are then inserted into the aneurysm to minimize blood flow into the aneurysm. As a result, blood clot formation is stimulated, and over time, fibrous tissue forms inside the aneurysm cavity, which leads to the complete blockage of the aneurysm. As illustrated in Fig. 1, the two treatment methods operate through distinct mechanisms: one involves direct surgical exposure and clipping of the aneurysm, while the other uses intravascular coil delivery. The coiling method is less invasive than surgical clipping and has the added benefit of acting as a scaffold, promoting tissue regeneration and potentially sealing the aneurysm permanently^4^. However, despite its advantages, coiling is associated with a significant recurrence rate, with up to one-quarter of treated aneurysms requiring retreatment^5^. Studies of human brain aneurysm samples, obtained from both surgical and autopsy specimens, have shown that sustained aneurysm repair depends on internal wound healing processes. These include connective tissue growth, fibroblast activity, collagen production, smooth muscle proliferation, capillary formation, and macrophage involvement^5–7^.

Although these biological processes are central to durable aneurysm repair, their evaluation in preclinical models has largely relied on qualitative or semi-quantitative histological assessment. Such approaches are inherently limited by observer variability, restricted scalability, and inconsistent criteria for defining tissue ingrowth across studies. As a result, comparing therapeutic strategies or systematically assessing the effects of pro-healing mediators remains challenging. Quantitative, reproducible measurement of intra-aneurysmal tissue formation is therefore a critical unmet need for understanding aneurysm healing dynamics, and for rigorously evaluating treatment efficacy in preclinical settings.

Building on earlier work by members of our team, who investigated aneurysm healing using mediators that promote tissue regeneration^6^, we applied machine learning (ML) models to detect and quantify tissue in growth in the aneurysm using the same dataset. Integrating biological insights with computational approaches offers a powerful way to improve aneurysm treatment by reducing rupture risk, minimizing recurrence, and limiting the need for retreatment. A critical step in optimizing such therapies is understanding the mechanisms that govern tissue ingrowth and aneurysm stabilization. Preclinical animal models, particularly murine systems, provide a controlled in vivo environment to explore these dynamics under conditions that approximate human biology. These models allow researchers to manipulate variables such as genetics, microenvironment, and aneurysm morphology to assess their impact on treatment outcomes. In one such model, monocyte chemotactic protein-1 (MCP-1), a cytokine involved in inflammation and tissue repair, was shown to play a pivotal role in driving intra-aneurysmal healing following coiling^6^. In this study, we employed the same murine model and applied advanced artificial intelligence (AI) techniques to quantitatively assess tissue ingrowth. By combining high-resolution histological analysis with deep learning, we aim to provide a reproducible framework for evaluating aneurysm healing and treatment efficacy.

In recent years, AI-driven models have received significant attention in biomedical research, particularly in medical imaging and histological image analysis. Advances in ML, particularly deep learning techniques such as convolutional neural networks (CNNs), have transformed the ability to interpret complex biological data with higher accuracy and efficiency than traditional manual or semi-quantitative methods. AI models have been effectively used in a wide range of applications, including tumor detection, organ segmentation, and tissue characterization^9–12^. These technologies have shown promise in automating tasks such as boundary segmentation, volumetric measurements, and feature recognition in medical images, including cardiovascular structure delineation, patient-specific hemodynamic modeling, and quantitative physiological parameter estimation, which were previously labor-intensive^13,14^. The use of AI and ML in the field of diagnosis, prediction, and treatment of brain aneurysms has gained considerable attention. Building on these advances, researchers employed a set of ML models^15^, including support vector machines, random forests, logistic regressions, and multilayer perceptron, to develop rupture-risk models for small aneurysms^16^. They included various patient-related features such as clinical data, comorbidities, alcohol intake, and smoking status alongside the aneurysm characteristics. These studies also investigated the hemodynamic parameters and found them to be the strongest predictors of aneurysm rupture. Another study employed a 3D CNN-based deep learning model to predict aneurysm rupture from plain magnetic resonance imaging (MRI) scans, including T1 sequences^17^. This method utilized 3D CNNs to automate the slice-to-slice and voxel-to-voxel feature extraction procedure. They employed an ensemble of three deep-learning models to automatically segment and detect cerebral aneurysms on MRI T1 images. The results were comparable to, or slightly better than, those obtained using computed tomography angiography (CTA) and magnetic resonance angiography (MRA), which are more generalized imaging techniques commonly employed by physicians for intracranial aneurysm segmentation, though they are not universally accessible.

ML models can predict the outcomes of aneurysm therapies, including endovascular coiling^18–22^. These models can predict whether a specific aneurysm treatment approach is optimal for an individual patient based on features such as aneurysm size, stent-assisted coiling (saccular), posterior circulation involvement, and other parameters. Ou et al.^18^ utilized a comprehensive set of pre-treatment features, including morphological characteristics from 3D digital subtraction angiography (DSA) images, clinical symptoms, blood test results, demographic information, medical history, lifestyle factors, and treatment details (e.g., treatment method, number of coils, stent metal coverage rate). They then applied a statistical model and ML models to predict the likelihood of complete occlusion following aneurysm treatment. Tian et al.^23^ developed ML models to predict periprocedural complications related to endovascular treatment for unruptured intracranial aneurysms. They also analyzed various clinical and morphological features, including patient age, gender, treatment modality, aneurysm size, location, shape, and several other parameters.

To our knowledge, this study presents a reproducible and fully automated histopathological image analysis framework for quantitative assessment of tissue ingrowth within cerebral aneurysms in a preclinical animal model. Intra-aneurysmal tissue formation is a key biological determinant of durable aneurysm healing, as it reflects progressive thrombus organization, connective tissue deposition, and cellular remodeling that stabilize the aneurysm sac following endovascular treatment. However, assessment of these processes has traditionally relied on qualitative or semi-quantitative histological evaluation, limiting reproducibility and cross-study comparability.

By employing deep learning-based segmentation on high-resolution histological images, our approach enables objective, reproducible quantification of both aneurysm sac morphology and intra-saccular tissue ingrowth. Unlike prior machine learning studies, which have predominantly utilized imaging modalities such as MRI, CT, or angiography, our methodology draws on the detailed structural information provided by histology, a less explored yet highly informative domain. This distinction is significant because histology provides direct insight into cellular and tissue-level changes, enabling more precise characterization of healing and tissue integration within aneurysm sacs. While conventional imaging captures macroscopic structural changes, it often lacks the resolution to detect subtle but critical histopathological indicators of aneurysm stability. Furthermore, the proposed model benefits from the incorporation of a boundary loss function^24^, enabling more precise detection of the boundaries in the stent-assisted-coiling area. By optimizing boundary recognition, the model achieves improved accuracy in segmenting regions of tissue ingrowth, which is critical for accurate quantification and analysis. Overall, this methodology addresses key limitations of manual histological analysis and establishes a standardized computational framework for evaluating biological stability and therapeutic efficacy in preclinical aneurysm models, with clear relevance for translational research aimed at reducing recurrence and improving long-term treatment outcomes. The main contributions of this study are fourfold:

1. We present an AI-based framework for accurate delineation of the aneurysmal saccular (or sac), which enables standardized definition of the biological domain in which healing and tissue remodeling occur following endovascular treatment.
2. We introduced a fully automated reproducible method for quantifying aneurysm sac area from histological images, providing an objective morphological metric relevant to aneurysm stability and treatment response.
3. We demonstrate a reproducible AI-based approach for quantification of tissue ingrowth, enabling precise assessment of the percentage of tissue ingrowth within the aneurysmal sac, a critical metric for assessing biological stabilization post-treatment.
4. By integrating sac morphology and tissue ingrowth analysis and validating performance through inter-observer agreement studies, our pipeline establishes a reproducible computational endpoint for evaluating therapeutic efficacy and biological healing in preclinical aneurysm models, supporting translational studies aimed at reducing recurrence risk.

## Results

### Machine learning pipeline overview

**Fig. 2:**
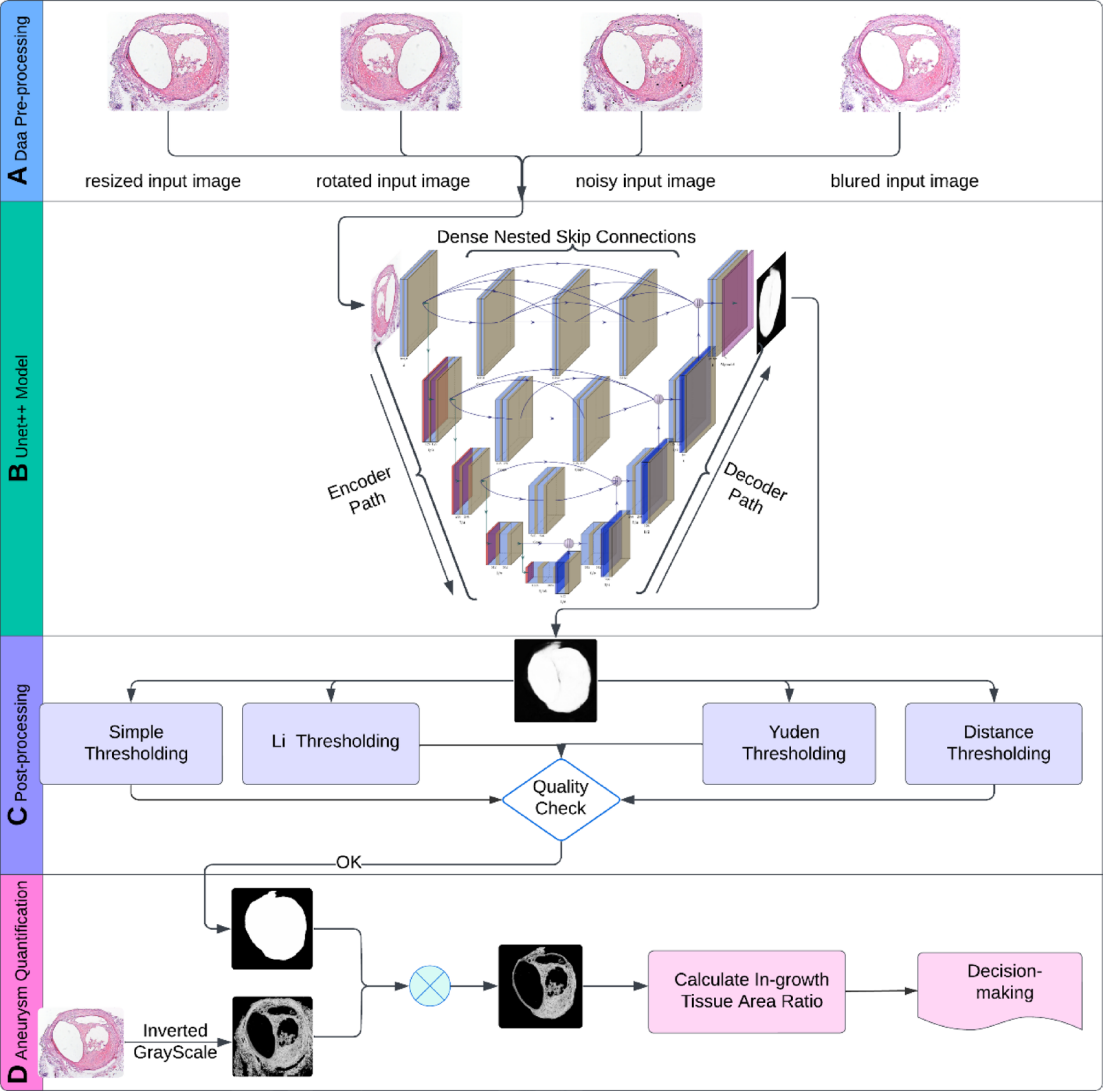
Schematic view of our intra-aneurysmal tissue ingrowth quantification pipeline. (**A**) The preprocessing step, including intensity enhancement and data augmentation, (**B**) The AI model architecture, in which the encoder path, decoder path, and dense nested skip connections of the Unet + + architecture are explicitly labeled (**C**) The post-processing step, including different thresholding techniques and finding the best among all, (**D**) The quantification step, using the original image and predicted saccular zone.

The proposed ML pipeline follows a structured workflow from data preprocessing to model deployment. The pipeline is designed to achieve accurate image segmentation, robust performance evaluation, and increased user accessibility through graphical user interface (GUI). The pipeline comprises seven key stages: data preprocessing, model training, post-processing, model evaluation, tissue ingrowth quantification, correlation analysis comparing AI-predicted measurements with human expert ratings, and deployment via a GUI. Figure 2 provides a visual summary of the workflow, with the subsequent steps outlined below.

1. Data preprocessing: The raw images of aneurysmal tissue ingrowth, originally sized at 2688 × 2200 pixels (corresponding to a physical area of approximately 5.9 mm² based on a spatial resolution of 1 μm² per pixel), were accompanied by ground truth image masks of the same dimensions, provided by neurosurgeons. These images underwent normalization and augmentation to enhance model generalization. The images were resized to 512 × 512 pixels to fit in the Unet + + architecture. On-the-fly data augmentation techniques, including rotation, flipping, and scaling, were employed to synthetically increase dataset size and variety. This approach presents the model with varied transformations of each image during training, effectively increasing the variability of training data. This step helps address the challenge of limited data availability and enhances the ability of the model to generalize^25–27^.
2. Segmentation model training: The preprocessed images are input into a Unet + + model to segment the aneurysmal sac region of the ingrowth tissue^28^.

**Fig. 3:**
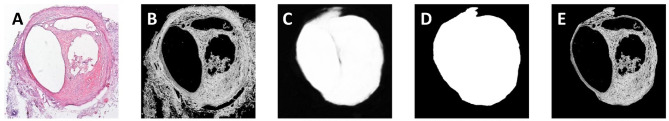
Postprocessing steps. (**A**) The original input image, (**B**) The inverted grayscale input image, (**C**) The predicted saccular zone image, (**D**) The binary mask of the predicted image, (**E**) The ingrowth tissue mask. The scalebar is 200 μm.

3. Postprocessing: Segmentation results from Unet + + are refined using several classical thresholding techniques^29^. For example, simple manual and adaptive methods such as Otsu’s thresholding^30^, Li’s minimum cross-entropy thresholding^31^, and techniques that determine the optimal threshold by minimizing the distance to the ideal point on the ROC curve (equivalently, maximizing Youden’s index)^32^ are employed to binarize probability maps and reduce noise. (Fig. 3)
4. Performance analysis: Segmentation performance is evaluated using the Dice coefficient, area under the receiver operating characteristic curves (AUC), precision, and recall. This analysis is conducted on a validation set.
5. Aneurysm quantification: After segmenting the aneurysm sac, tissue ingrowth regions are identified using the binary segmentation masks and the grayscale version of the input image. This process yields a percentage measurement of tissue growth within the sac boundary.
6. Inter-observer agreement: The consistency between tissue ingrowth measurements obtained by our AI pipeline and those by neuropathologists was assessed using Cohen’s κ (kappa) statistic^33^, evaluating the accuracy and reliability of the measurements.
7. End-user GUI: Our pipeline is presented as an intuitive, user-friendly GUI that allows users to upload new images, view segmentation outcomes, and interpret the results with ease.

### Unet + + Segmentation Model and Training

Our segmentation pipeline is based on the Unet + + architecture^28^, which extends the traditional U-Net model^34^to enhance segmentation performance. Unet + + is characterized by nested dense skip connections and deep supervision, which improve the model’s ability to capture fine-grained details while preserving contextual information. This architecture resembles a U-shaped structure where the contracting path functions as an encoder, and the expansive path serves as a decoder^28^. The contracting path captures context and extracts features, while the expansive path reconstructs the segmentation map.

In this study, we utilized a pretrained ResNet-50 model as the encoder^35^, incorporating its deep residual learning capabilities for enhanced feature extraction. By utilizing ResNet-50 as the encoder, we leverage the parameters of a pretrained model that was trained on large image datasets, such as ImageNet^36^, which provides a better initialization for training, especially when working with a small dataset. The residual connections in ResNet-50 facilitate efficient gradient flow, which improves the model’s ability to learn complex representations. Moreover, Unet + + enhances the traditional skip connections by introducing nested skip connections that allow for multiple levels of feature fusion. This process enables the model to merge high-level semantic information from deeper layers with fine-grained spatial details from shallower layers more effectively^28^. The architecture of the Unet + + segmentation model is illustrated in Fig. 2B, highlighting the encoder layers, dense nested skip connections between intermediate encoder and decoder layers, and the final output layer that generates the predicted saccular zone.

The training approach employs a supervised learning paradigm where the model predicts continuous grayscale intensity heatmaps instead of discrete binary masks for segmentation. By predicting smooth intensity values, the model can capture subtle gradations within the images more effectively than with conventional binary mask predictions, resulting in higher accuracy. To achieve the best performance, we use the mean squared error (MSE) loss function defined in Eq. 1, which quantifies the average squared difference between predicted and ground truth intensities:

1 $${\mathcal{L}}_{MSE}=\frac{1}{N}\sum_{i=1}^{N}{\left({y}_{i}-{\widehat{y}}_{i}\right)}^{2},$$

where ${y}_{i}$ and ${\widehat{y}}_{i}$ represent the true and the predicted intensity at pixel $i$, and $N$ is the total number of pixels. This approach preserves the granularity of grayscale intensities, which is essential for capturing fine-grained structural details and improves segmentation performance by highlighting subtle variations. Consequently, our method provides more reliable and robust segmentation of saccular aneurysm boundaries.

### Postprocessing Analysis

Figure 3illustrates the post-processing steps applied to the output of the Unet + + segmentation model^28^. Specifically, the model generates a continuous grayscale heatmap (Fig. 3C), with the output values are normalized to the range $[0,1]$using a sigmoid activation function^37^. Then, a threshold value within the range of $[0,1]$ is applied to the heatmap to generate a binary aneurysm sac mask (Fig. 3D). Motivated by the comparative analysis of thresholding approaches in remote sensing image change detection by Xing et al.^38^., we evaluated several thresholding techniques. We implemented two thresholding strategies:

1. *Manual thresholding*: Applying fixed cutoffs (e.g., 0.1, 0.5, or 0.8) uniformly across all predicted heatmaps.
2. *Adaptive thresholding*: Computing an optimal threshold for each predicted heatmap based on its intensity distribution or ROC characteristics. We employ:

- Otsu’s method^30^, which maximizes between-class variance.
- Li’s index^31^, which minimizes cross-entropy between foreground and background.
- Yuden’s J statistics defined in Eq. 2^32^, selecting.

2 $${\tau}_{\mathrm{Y}\mathrm{o}\mathrm{u}\mathrm{d}\mathrm{e}\mathrm{n}}=\underset{\tau}{\mathrm{arg\:min}}\left(TPR\left(\tau\right)-FPR\left(\tau\right)\right)$$.

- Distance-based thresholding defined in Eq. 3, selecting.

3 $${\tau}_{\mathrm{D}\mathrm{i}\mathrm{s}\mathrm{t}\mathrm{a}\mathrm{n}\mathrm{c}\mathrm{e}}=\underset{\tau}{\mathrm{arg\:min}}\sqrt{{FPR\left(\tau\right)}^{2}+{\left(1-TPR\left(\tau\right)\right)}^{2}}$$.

**Fig. 4:**
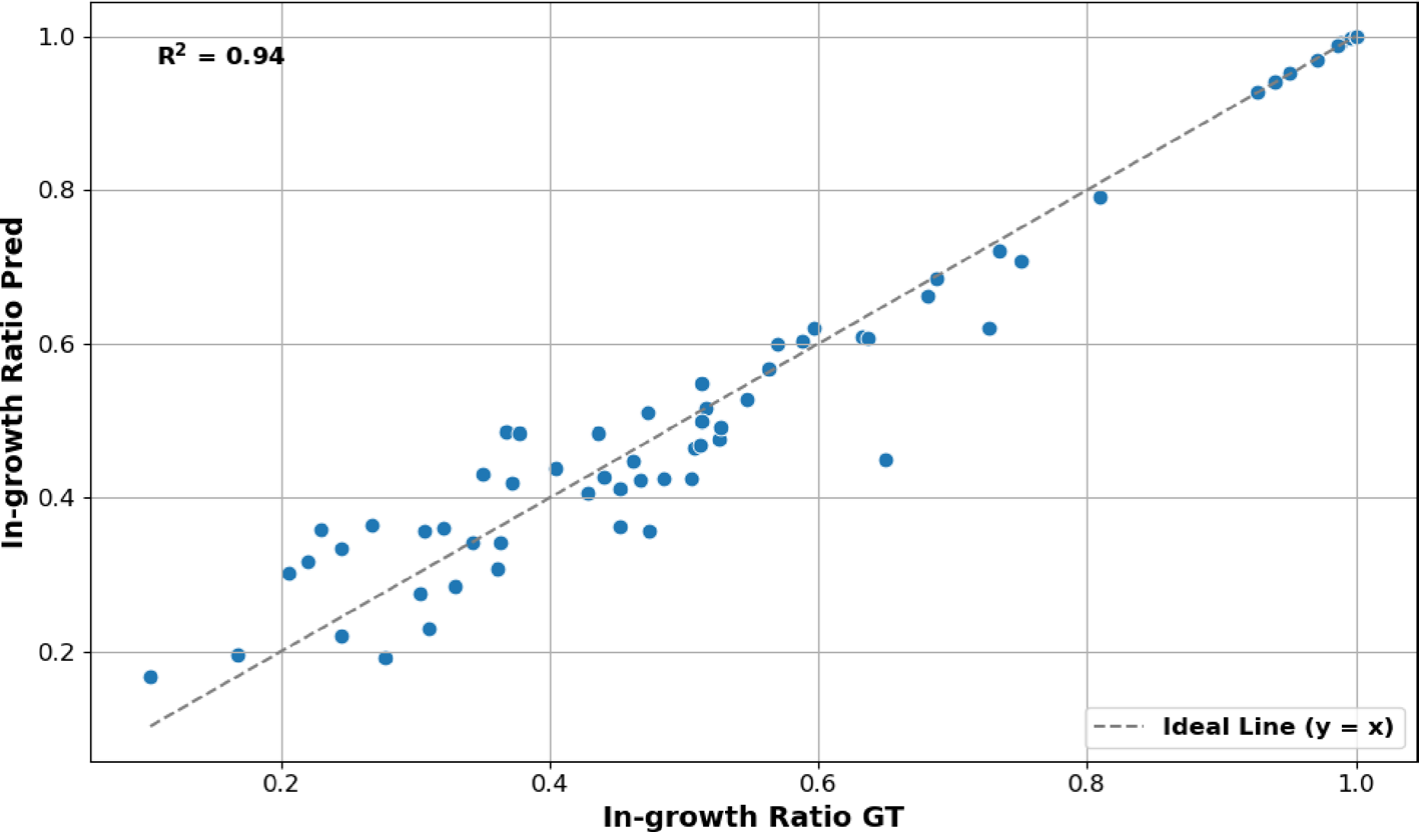
Scatter plot comparing predicted ingrowth ratio with ground truth (GT) measurements. Each point represents a sample image. The dashed gray line indicates the ideal correspondence ($y=x$) between predictions and GT. The coefficient of determination (${R}^{2}=0.94$) indicates a strong agreement, suggesting high model accuracy in estimating ingrowth ratios.

The thresholding approaches used here refine the segmentation results by distinguishing between tissue structures from the background (Fig. 3D). Subsequently, the estimated binary mask is applied in a scalar product operation with the inverted grayscale input image (Fig. 3B) to extract the ingrowth tissue regions (Fig. 3E). The grayscale image is inverted to ensure that the background and foreground pixels have values of 0 and 1, respectively, aligning with the mask representation where background pixels are 1 and foreground pixels are 0. To quantify the ingrowth tissue, we computed the ingrowth ratio, defined as the number of ingrowth tissue pixels relative to the estimated saccular zone area. Figure 4 depicts the scatter plot comparing the predicted ingrowth ratios with the corresponding ground truth values across individual images. The close alignment of points along the identity line and a high coefficient of determination (${R}^{2}=0.94$) indicate strong agreement between the predicted and ground truth ratios. This metric provides clinicians with a quantitative assessment of the treatment’s performance, supporting more informed clinical decisions.

### Performance Evaluation of the Machine Learning Pipeline

To evaluate the model’s reliability and generalizability, we implemented a 10-fold cross-validation strategy and applied multiple evaluation metrics to assess segmentation quality^15^. We randomly partitioned the dataset into 10 equal parts or *folds*. In each iteration, we trained the model on nine folds and validated the remaining one, ensuring that each fold serves as the validation set exactly once. Averaging the results across all folds, this approach yields a robust and stable estimate of the effectiveness of the model, minimizing the impact of random variation and reducing the risk of overfitting associated with a single train-test split. This comprehensive evaluation strategy allows us to assess the ability of the model to generalize to unseen data.

We evaluated the segmentation results using several metrics. The Dice coefficient measures the overlap between the predicted segmentation mask and the ground truth, and effectively addresses class imbalance issues common in medical image segmentation^39^. Accuracy quantifies the overall proportion of correctly classified pixels. Recall assesses the model’s ability to detect all relevant regions, while precision measures its ability to minimize false positives. Specificity measures the model’s ability to correctly identify non-relevant regions. The area under the ROC curve (AUC) serves as a comprehensive metric for evaluating the discriminatory power of the model across a range of threshold values, which is especially relevant given that our outputs are grayscale intensities ranging from 0 to 1.

Each intensity value can be interpreted as a probability, representing the likelihood that a given pixel belongs to the aneurysm sac. By applying various threshold levels to these continuous outputs, we binarized the predictions, enabling the calculation of true positive and false positive rates at each threshold. Additionally, we computed the MSE to assess the average squared difference between predicted and actual values, which offers insights into prediction accuracy. Throughout training, we monitored learning curves, including training and validation loss curves, to detect overfitting or underfitting. This monitoring guided the adjustment of hyperparameters such as learning rate, batch size, and number of epochs to optimize model performance. Moreover, we evaluated the performance of the model on successive iterations to assess stability and convergence trends. Table 1 presents our key performance metrics, including average Dice coefficient, accuracy, recall, precision, specificity, AUC, and MSE, calculated as averages across unseen validation datasets in a 10-fold cross-validation setting. This table provides a clear overview of each metric, facilitating evaluation of segmentation performance and identification of areas needing improvement. A high Dice coefficient and AUC indicate robust overlap and strong discriminatory ability, respectively; high specificity and precision provide insight into the model’s reliability in terms of minimal false positives and correctly identified non-relevant regions. Low MSE values indicate minimal differences between the predicted and ground truth masks.

**Table 1:** Average comparison results of several metrics (in percentages) for aneurysmal sac zones and ingrowth tissue predictions. Note: The largest value in each column is **bold faced**, and the second largest value is *underlined*.

Threshold	Aneurysmal sac zone metrics					
	Dice	Accuracy	Sensitivity	Specificity	AUC	MSE
**Manual (0.1)**	93.04	95.82	*97.60*	**94.42**	99.23	0.2660
**Manual (0.5)**	94.56	**96.93**	94.19	94.31	*99.24*	0.2660
**Manual (0.8)**	93.92	96.64	91.42	94.22	**99.25**	0.2660
**Otsu**	*94.57*	**96.93**	94.19	94.30	*99.24*	0.2660
**Li**	**94.58**	*96.88*	95.67	94.36	*99.24*	0.2660
**Youden**	91.34	94.51	**98.51**	*94.41*	99.22	0.2660
**distance**	93.51	96.20	*97.60*	94.37	99.22	0.2660

Threshold	Ingrowth tissue metrics					
	Dice	Accuracy	Sensitivity	Specificity	AUC	MSE
**Manual (0.1)**	93.04	95.83	97.60	64.98	96.27	0.0417
**Manual (0.5)**	**95.23**	*97.71*	94.89	*66.22*	*96.78*	*0.0229*
**Manual (0.8)**	*95.18*	**99.03**	93.79	**66.31**	95.96	**0.0097**
**Otsu**	**95.23**	*97.71*	94.88	*66.22*	*96.78*	*0.0229*
**Li**	94.76	97.08	95.89	65.84	96.71	0.0292
**Youden**	91.35	94.62	**98.33**	64.18	95.43	0.0538
**distance**	93.80	96.52	*97.79*	65.26	**96.79**	0.0348

**Fig. 5:**
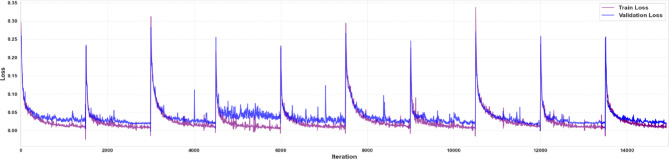
Loss curves. Training and validation loss curves for all 10 folds during 10-fold cross-validation, illustrating the model’s performance across the folds.

Figure 5 shows the evolution of training and validation loss across the iterations for all ten cross-validation folds, allowing assessment of model convergence. A continuous downward trend in both curves across all 10 folds indicates successful learning. Divergence between the training and validation loss trajectories would indicate the need to adjust training strategies – such as applying a learning-rate scheduler, modifying batch size, or increasing regularization strength – to restore alignment and ensure proper convergence.

**Fig. 6:**
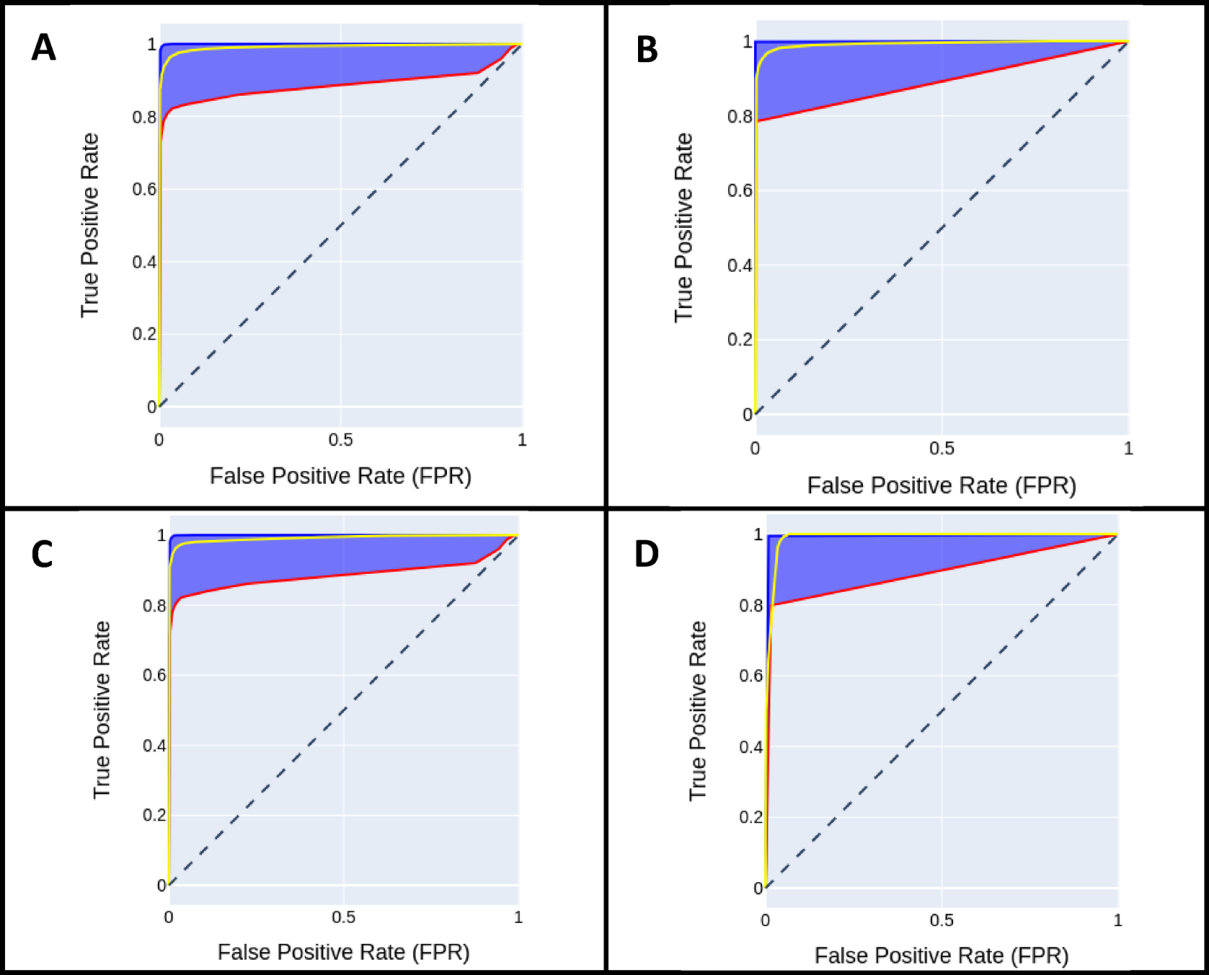
ROC curve plots for aneurysmal sac zones and ingrowth tissue predictions applying different thresholding techniques. (**A**) ROC curve plots for aneurysmal sac zone prediction using the manual threshold value equal to 0.5, (**B**) ROC curve plots for ingrowth tissue prediction using manual threshold value equal to 0.5, (**C**) ROC curve plots for aneurysmal sac zone prediction using Li’s thresholding method, (**D**) ROC curve plots for ingrowth tissue prediction using Li’s thresholding method. Note: The yellow plots refer to the average values, the red plots refer to minimum values, and the blue plots refer to maximum values of the TPRs and FPRs pairs. In addition, the blue shaded regions refer to the area between minimum and maximum values.

Figure 6 depicts the ROC curves, showing true positive rates versus false positive rates for both the optimal thresholding method (based on the best Dice coefficient) and the simple thresholding method with a fixed threshold of 0.5. This plot provides a nuanced view of model performance across a wide range of decision thresholds, offering deeper insights beyond the AUC into the model’s ability to generalize across varying confidence levels.

In addition to quantitative metrics, we conducted qualitative visual comparisons by presenting predicted segmentation masks alongside ground truth masks. These visualizations demonstrated the model’s ability to accurately delineate aneurysmal regions and capture fine structural details through its grayscale intensity outputs. The model exhibited robust performance, as supported by consistent results in both quantitative and qualitative analyses. This reliability underscores its potential clinical utility in providing accurate segmentation of aneurysmal structures, thereby assisting clinicians in assessing tissue ingrowth and informing treatment decisions.

**Fig. 7:**
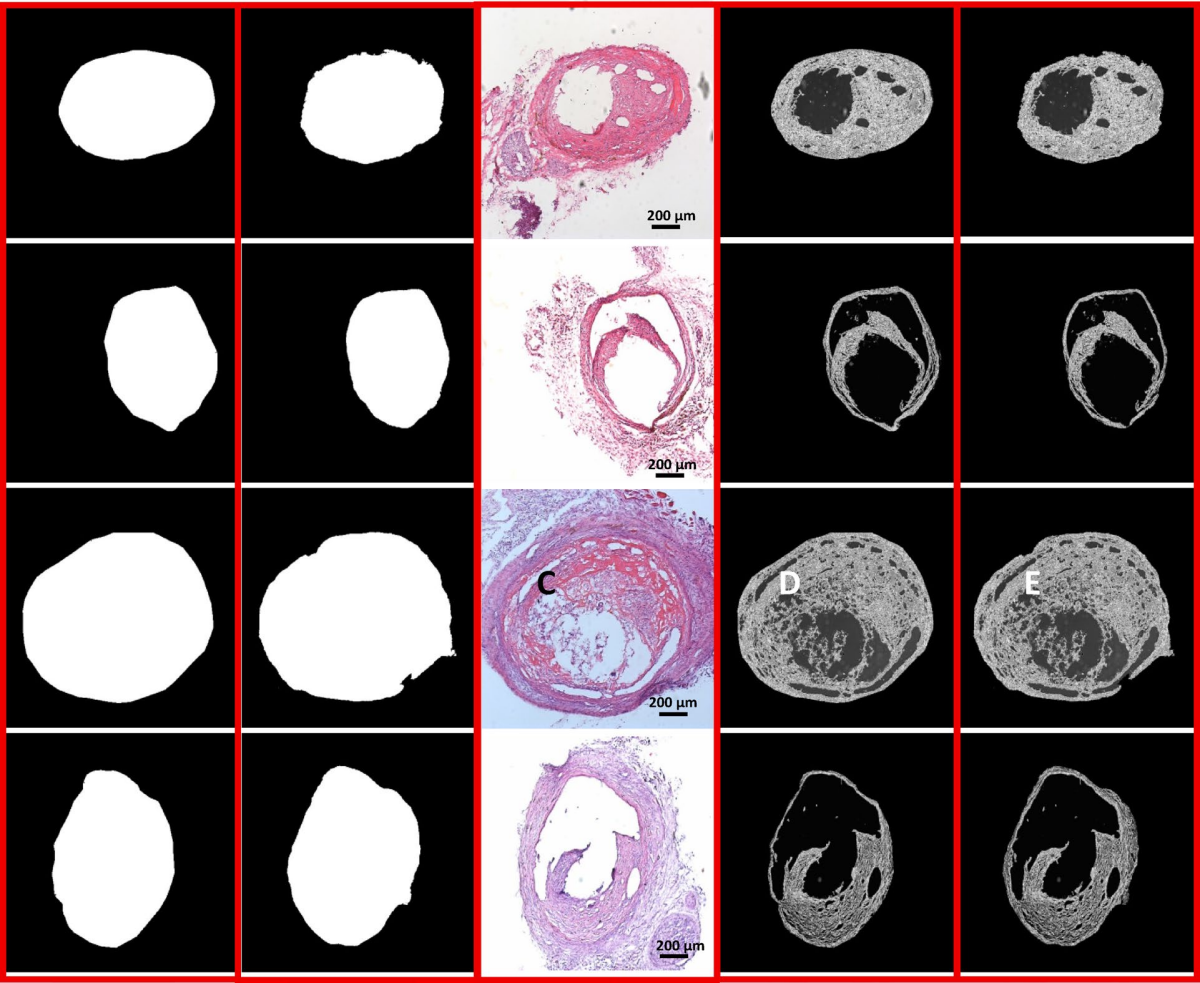
Representative sample images illustrating saccular zone and ingrowth tissue predictions alongside their ground truths. (**A**) Ground truth saccular zone images, (**B**) Predicted saccular zone images, (**C**) Original images of aneurysmal ingrowth tissue, (**D**) Ground truth ingrowth tissue, (**E**) Predicted ingrowth tissue. The scalebar is 200 μm.

Figure 7 presents representative examples, displaying the original images (7 C), ground truth saccular regions (7 A), the model’s predicted segmentations (7B), and the corresponding ingrowth tissue images (7D, 7E). Each row corresponds to a specific case, showing the reference image, ground truth mask, and the model’s segmentation output. These examples further highlight the model’s effectiveness in capturing subtle structural features within aneurysmal sacs. Visual inspection of these results supports the conclusion that the proposed pipeline reliably distinguishes regions of interest from the background.

**Fig. 8:**
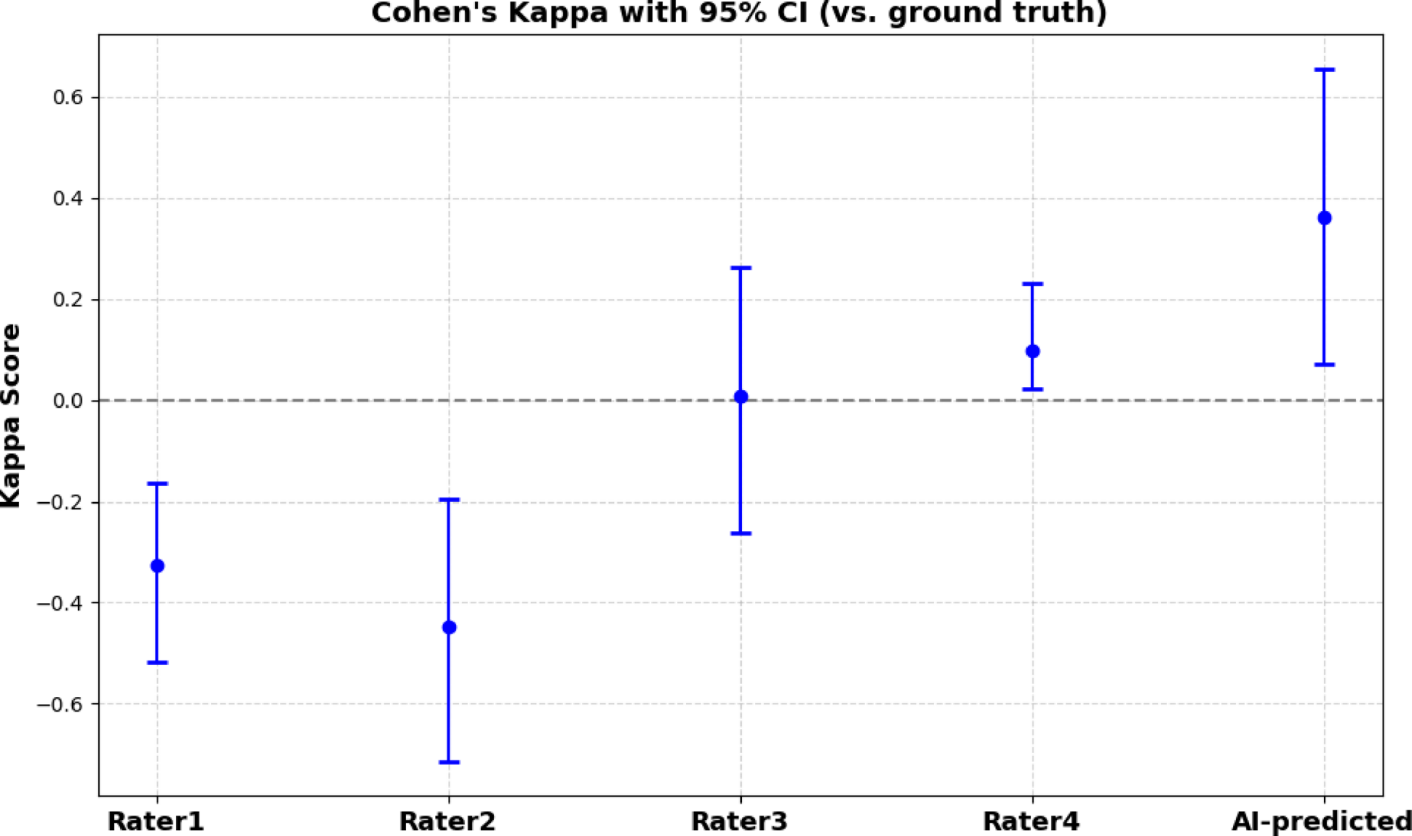
**Cohen’s κ agreement scores** with 95% confidence intervals between trained raters and the expert neurosurgeon (ground truth) for tissue ingrowth quantification across 32 blinded images.

### Tissue ingrowth measurement and AI-pathologist agreement

We recruited students who were rigorously trained under the supervision of expert neurosurgeons to ensure accurate and consistent measurements of tissue ingrowth. These students followed a standardized, blinded protocol and used ImageJ software to visualize and quantify the tissue ingrowth, ensuring consistency across evaluations. The protocol involved outlining the original lumen and the newly formed lumen area on tissue images, measuring both regions, and recording the values in a structured format for subsequent analysis.

To assess agreement between manual and automated quantification of tissue ingrowth, we compared the binarized assessments from four trained raters and the AI-predicted outputs generated by our Unet + + model against the expert neurosurgeon’s ratings, which served as the ground truth. This evaluation was conducted on a blinded dataset comprising 32 histological images that were entirely independent from the datasets used for training and testing the Unet + + model. We used Cohen’s κ statistic^33^ to assess agreement at the whole-slide pixel level. We computed 95% confidence intervals for each Cohen’s κ score using bootstrap resampling (1,000 iterations), allowing us to quantify the uncertainty around agreement estimates robustly. The resulting Cohen’s κ values and their confidence intervals are visualized in Fig. 8, highlighting the degree of agreement between each rater, including the AI-predicted output, and the expert neurosurgeon. Notably, the AI model achieved the highest *κ* score, indicating the strongest concordance with the expert annotations among all raters. This focused comparison, centered on agreement with the expert rather than pairwise rater comparisons, highlights the relative performance and reproducibility of each method for tissue ingrowth quantification, underscoring the potential of AI to provide consistent and expert-aligned assessments.

### End-user graphical user interface (GUI)

We developed an end-user plugin using HistomicsUI^40,41^ within Digital Slide Archive (DSA, https://athena.rc.ufl.edu/), an application programming interface of the DSA for running Python codes to predict ingrowth tissue and saccular zone for a given aneurysmal tissue image. DSA is an open-source, cloud-based histology management system^40,41^ where custom deep learning-based histology analysis workflows can be deployed as individual pipelines^42,43^or integrated as part of a plugin suite^44^. The HistomicUI interface displays the detected saccular zone and tissue in growth over the original image, enabling users to compare and analyze the results visually.

**Fig. 9:**
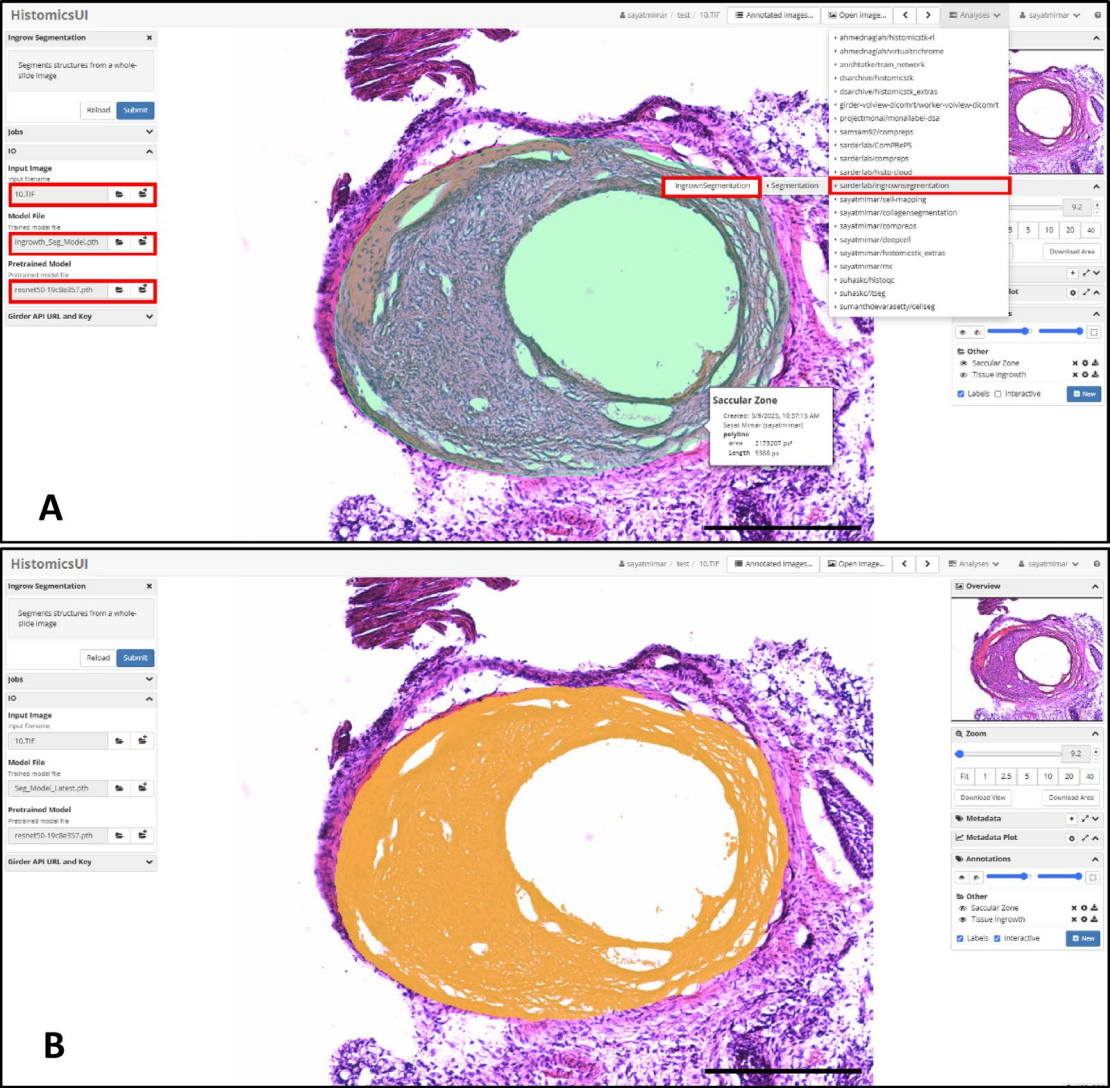
Screenshot of the cloud-based graphical user interface (GUI) for saccular zone and tissue ingrowth detection. The panel shown in (A) presents the ingrown segmentation plugin within HistomicsUI. The user selects the input image and the model files. Once selected, the user clicks the submit button and the segmentation job starts to run. The output masks for saccular zone (A) and tissue ingrowth (B) are shown on the histology crop once the job is completed. By hovering the mouse over the saccular zone, the users can see the structural information (length and area) about the zone. The scalebars correspond to 135 μm.

The workflow and user interface of the plugin are illustrated in Fig. 9, with each panel depicting a specific aspect of the process. Panel A displays the plugin integrated within HistomicsUI, where users can launch a job by selecting an input image and a pretrained segmentation model, both publicly available in DSA. The model is located under Collections at the path: models/segmentation_models/Ingrowth_segmentation. Panel B presents the resulting segmentation overlay, highlighting ingrowth tissue and displaying key morphological measurements, such as saccular zone area, tissue area, and length, which appear interactively when hovering over corresponding regions. The interface also allows users to edit or refine the saccular zone and tissue ingrowth boundaries using intuitive interactive tools, enabling greater control over the segmentation results. For a more comprehensive demonstration of the GUI’s features, please refer to Supplementary Figs. 1–3.

## Discussion

Manual segmentation and quantification of tissue ingrowth in cerebral aneurysm histology are critical but labor-intensive and subject to significant inter-observer variability. Recent studies have explored automated techniques for vascular image analysis, focusing primarily on clinical imaging modalities or general histopathology^45,46^. However, applications to preclinical aneurysm models remain limited, with most methods constrained by modest accuracy or insufficient validation. To our knowledge, there are no reports of deep learning pipelines delivering reproducible segmentation and quantification of both aneurysm sac boundaries and intra-aneurysmal tissue ingrowth in preclinical histological images. From a biological standpoint, intra-aneurysmal tissue ingrowth reflects the degree of thrombus organization, connective tissue deposition, and cellular remodeling that underlie durable aneurysm healing. Reliable quantification of these processes is therefore essential for mechanistic evaluation of therapeutic strategies aimed at reducing aneurysm recurrence and improving long-term stability.

In this context, our study presents a comprehensive machine-learning pipeline for segmentation and quantification of saccular aneurysm zones and associated tissue ingrowth, achieving state-of-the-art performance in preclinical cerebral aneurysm models. Moreover, the ability to accurately quantify tissue ingrowth provides an objective surrogate marker of aneurysm healing, enabling direct comparison of biological response across treatment conditions and experimental cohorts. Strong agreement between predicted and ground truth ingrowth ratio values, evidenced by the tight clustering along the identity line in the scatter plot, demonstrates the model’s ability to accurately capture both overall aneurysm structure and finer-grained ingrowth regions (Fig. 4).

Quantitatively, our Unet + + architecture performs strongly, with average Dice coefficients of 94.6% for sac segmentation and 95.2% for ingrowth tissue identification, alongside AUCs exceeding 99% and 96%, respectively (Table 1). Low mean squared error and high specificity further indicates that our model not only delineates regions of interest with precision but also effectively suppresses false positive detection of tissue ingrowth, which is critical for avoiding overestimation of healing and misinterpretation of therapeutic efficacy.

Loss-curve analysis across all ten cross-validation folds reveals smooth and convergent training dynamics, with no sign of overfitting (Fig. 5). Nevertheless, given the relatively small and homogeneous dataset, some degree of model optimism cannot be fully excluded despite the use of cross-validation, data augmentation, and regularization strategies. Accordingly, the present results should be interpreted as demonstrating the feasibility and robustness of the proposed analysis pipeline rather than definitive evidence of broad generalizability. This stability is attributable to our rigorous preprocessing steps (augmentation, normalization). In our threshold-sensitivity analysis (Fig. 6), both manual ($0.5$) and adaptive (Li’s) thresholding yielded AUCs $>0.95$ for sac segmentation and $>0.90$for ingrowth detection, with Li’s method offering marginally higher sensitivity at the expense of specificity. These findings align with prior histopathological image analysis studies demonstrating that adaptive thresholding methods are more robust to staining variability and intensity heterogeneity than fixed global thresholds, particularly in complex biological tissues^47–49^. From a biological perspective, this robustness to staining heterogeneity is particularly important in histopathological analysis, where variability in tissue preparation and contrast can otherwise confound quantitative assessment of healing.

Qualitative evaluation of representative cases (Fig. 7) demonstrates that the model accurately delineates both the saccular boundary and the ingrowth region, even in anatomically complex or low-contrast scenarios. Quantitatively, the AI-predicted assessments achieved the highest Cohen’s κ score in comparison to all human raters when benchmarked against the expert neurosurgeon, indicating superior alignment with the ground truth. This level of agreement places the model’s performance well within the range of inter-observer variability and underscores its potential to deliver consistent, expert-level assessments—ultimately reducing the burden of manual annotation in large-scale tissue ingrowth analysis. By reducing observer-dependent variability, the proposed framework enables more consistent longitudinal and cross-study comparisons of aneurysm healing, which is essential for evaluating subtle biological effects of pro-healing interventions.

Despite promising results, this study has important limitations related to dataset size and diversity. The dataset comprises 64 histological slides derived from a single preclinical aneurysm model, which may limit generalizability across species, disease states, or staining protocols. While our use of data augmentation, transfer learning, and cross-validation mitigates overfitting risk, these strategies do not substitute for external validation on independent datasets. Future studies incorporating multi-institutional datasets and external validation cohorts will be essential to establish the broader applicability of the proposed framework. Additionally, we plan to integrate an adaptive learning-rate scheduler and dynamic batch-size adjustment into the training process to further optimize convergence and model stability. Finally, incorporating active-learning strategies, where the model solicits expert annotations for uncertain regions, could enhance performance in edge-case scenarios. While murine models provide controlled insight into aneurysm healing mechanisms, future validation on human aneurysm specimens will be necessary to establish clinical translatability and to assess robustness across diverse pathological contexts.

To facilitate broader adoption, we deployed the full pipeline within an intuitive, cloud-based graphical user interface. This tool enables non-technical users to upload images, visualize segmentation overlays, refine boundaries interactively, and export quantitative metrics. By automating and standardizing histological assessment of aneurysm healing, this platform enables scalable, reproducible evaluation of endovascular therapies in preclinical studies, supporting data-driven optimization of treatment strategies prior to clinical translation.

## Materials and methods

### Animal model and aneurysm characteristics

This study utilized a well-established murine model to investigate the development and treatment of carotid aneurysms under controlled experimental conditions^6^. Both female and male C57BL/6 mice (8–12 weeks, 20–25 g; Charles River, Massachusetts, USA) were utilized for these experiments. To induce aneurysms in a murine model, mice were anesthetized using isoflurane. A midline neck incision was made to isolate the right common carotid artery (RCCA), which was then exposed to elastase (10 U/mL in phosphate-buffered saline [PBS]; Worthington Biochemical Corporation, New Jersey, USA) for 20 min. Following elastase exposure, distal occlusion was performed using cauterization, resulting in the formation of saccular aneurysms. Three weeks after the aneurysm formation, treatment was administered by placing platinum coils into the aneurysms. The coils were coated with either a CXCL1 antibody (100 µg; R&D Systems, Minnesota, USA) or an isotype-matched IgG control (100 µg; R&D Systems, Minnesota, USA). This ensured a consistent method of aneurysm induction and treatment across the experimental groups.

### Ethics approval statement

All experimental protocols were approved by the University of Florida Institutional Animal Care and Use Committee (UF IACUC), under protocol number IACUC202200000156.

### Animal use compliance statement

All methods were carried out in accordance with relevant guidelines and regulations, including those set forth by the University of Florida and UF IACUC.

### Compliance with ARRIVE guidelines

This study is reported in accordance with the ARRIVE (Animal Research: Reporting of In Vivo Experiments) guidelines (https://arriveguidelines.org).

### Tissue preparation & staining

Tissues were harvested and processed carefully to preserve the structural integrity of the aneurysms and surrounding regions:

1. Euthanasia and Perfusion: The mice were euthanized by cardiac perfusion with 4% paraformaldehyde (PFA) (Santa Cruz Biotechnology, Texas, USA). This step ensured proper fixation and preservation of tissue architecture.
2. Tissue Harvesting: The RCCA was carefully excised, immersed in 4% PFA overnight, and subsequently transferred to 18% sucrose for cryoprotection over an additional 24-hour period.
3. Cryosectioning: The tissues were frozen in Optimal Cutting Temperature (OCT) compound using a combination of dry ice and 2-methylbutane, allowing for cryosectioning. Thin sections of 5 μm thickness were prepared to facilitate histological staining and imaging.
4. Histological Staining: The tissue sections were stained using hematoxylin and eosin (H&E) to visualize cellular and tissue-level changes. This staining method provided clear contrast for assessing structural changes in the aneurysm and surrounding tissue.

### Imaging

We collected high-resolution histological images of aneurysmal sacs from preclinical models at the Neurosurgery Department, University of Florida College of Medicine, using an Olympus IX71 fluorescent microscope (Olympus America, Center Valley, Pennsylvania) at 10× magnification. The dataset comprises 64 brightfield whole slide images (WSIs), each representing a distinct mouse from a cohort of over 50 animals. For consistency and quality, one high-quality hematoxylin and eosin (H&E) stained image was selected per mouse. The spatial resolution of the H&E images is approximately 1 μm per pixel, providing sufficient detail for microscopic analysis of tissue morphology. These images provide a detailed histological perspective of aneurysmal tissues, essential for pathology studies and morphological analyses, particularly in the context of tissue ingrowth formation following treatment. Quantification of tissue ingrowth was performed using Image Pro software (Media Cybernetics, Maryland, USA), ensuring high-resolution measurements of structural changes within the aneurysmal sac.

To evaluate inter-rater reliability, a subset of the dataset was used for an independent rater study. Each rater assessed one representative image per mouse. The raters involved in this study were trained student researchers who manually annotated both the original (pre-treatment) and newly formed lumens within the aneurysmal sac.

### Machine learning pipeline development

We trained the Unet + + deep CNN model^28^ to predict the ingrowth of tissue in the saccular zone of the aneurysms. Unet + + is designed to capture fine details and maintain spatial information in segmentation output, which is essential for accurately delineating complex cerebral aneurysm structures.

Given the limited size of our dataset, we adopted a transfer learning approach by initializing the encoder of the Unet + + architecture with a Resnet-50 model^35^pretrained on ImageNet^36^. The ResNet-50 encoder comprises 16 residual blocks organized across four stages, forming a 50-layer deep network^35^. Each residual block incorporates identity shortcut connections that help preserve gradient flow, thus addressing the vanishing gradient problem typically encountered in deep architectures. Unet + +^28^as an enhancement of the conventional U-Net^34^, introduces a nested skip-connection design that densely links encoder and decoder sub-networks across multiple levels. This configuration promotes richer multi-scale feature aggregation and improved segmentation precision. The model was trained end-to-end using this architecture, without further structural modification.

### Model training and validation

A 10-fold cross-validation approach was employed to train and validate the model, ensuring robust and generalizable results^15^. The dataset was split into 10 folds, with each fold used once as a validation set and the remaining nine as training data. This process was repeated so that each data point was used for validation exactly once across the 10 iterations. The use of this method contributes to the avoidance of overfitting and allows us to complete evaluation of the performance of the model. Data augmentation techniques were applied to increase variability in the training set and improve the model’s generalization to unseen data. Augmentations such as rotation, scaling, and flipping were applied to synthetically increase training set size and variability. The model was optimized using mean squared error loss, which is effective in segmentation tasks and explicitly maximizes alignment between predicted and ground truth masks. The Adam optimizer was used for training, with an initial learning rate of 0.001. If the validation loss did not improve after 10 consecutive epochs, the learning rate was reduced by a factor of 0.1.

### GUI implantation in digital slide archive

Users can log in to the Digital Slide Archive (DSA; https://athena.rc.ufl.edu/) as a public user with the credentials: username: public; password: public. Whole-slide images (WSIs) for analysis can be uploaded to the Public or Private folders under the public user account. The WSI can be visualized in HistomicUI. Users can run the plugin from the path Analyses/sarderlab/ingrownsegmentation/Segmentation. The segmentation algorithm is packaged as a Docker image (Docker Inc., Palo Alto, CA), a framework that enables users to build and run containerized applications. The generated container follows the Slicer CLI workflow interface, which allows HistomicsUI to display an intuitive browser-based user interface to adjust algorithm parameters and execute the program. AI generated annotations are overlaid on the input image in JavaScript Object Notation (JSON) format and displayed in HistomicsUI as annotation layers.

### Code and data sharing

The Code for the segmentation model plugin is available at the GitHub repository: https://github.com/SarderLab/Ingrowth-Segmentation-DSA-Plugin. The datasets used and analyzed during the current study are available from the corresponding author on reasonable request.

## Supplementary Information

Below is the link to the electronic supplementary material.

Supplementary Material 1

## Data Availability

The Code for the segmentation model plugin is available at the GitHub repository: https://github.com/SarderLab/Ingrowth-Segmentation-DSA-Plugin. The datasets used and analyzed during the current study are available from the corresponding author on reasonable request.

## Declarations

### Supplemental Information

Supplementary Materials.docx.
