# Supplementary material for: A Machine learning pipeline to investigate tissue ingrowth in cerebral aneurysms using preclinical animal models
Source: Sci Rep. 2026 Mar 13;16:13352. doi: 10.1038/s41598-026-43798-w (PMC13106824; doi:10.1038/s41598-026-43798-w)
Supplement: Supplementary file 1 — Supplementary Material 1 [file 41598_2026_43798_MOESM1_ESM.docx]

**Supplementary Materials**

Afsari *et al. “*A Machine Learning Pipeline to Investigate Tissue Ingrowth in Cerebral Aneurysms Using Preclinical Animal Models*”*

**Table Of Contents**

**Navigating Digital Slide Archive and running the pipeline in the End-User GUI. 1**

**Navigating Digital Slide Archive and running the pipeline in the End-User GUI**

A user can log in to the Digial Slide Archive (DSA; https://athena.rc.ufl.edu/) as a public user with the following credentials: Username: public; password: public. The user can upload an image (see **Supp. Fig. 1**) in a folder. The image can be visualized in the graphical user interface by clicking the “Open in HistomicsUI” button at the top right (see **Supp. Fig. 2)**. Upon opening the WSI in the HistomicsUI viewer, the user can zoom in and out and hover over regions in the slide to visualize different structures at fine detail.


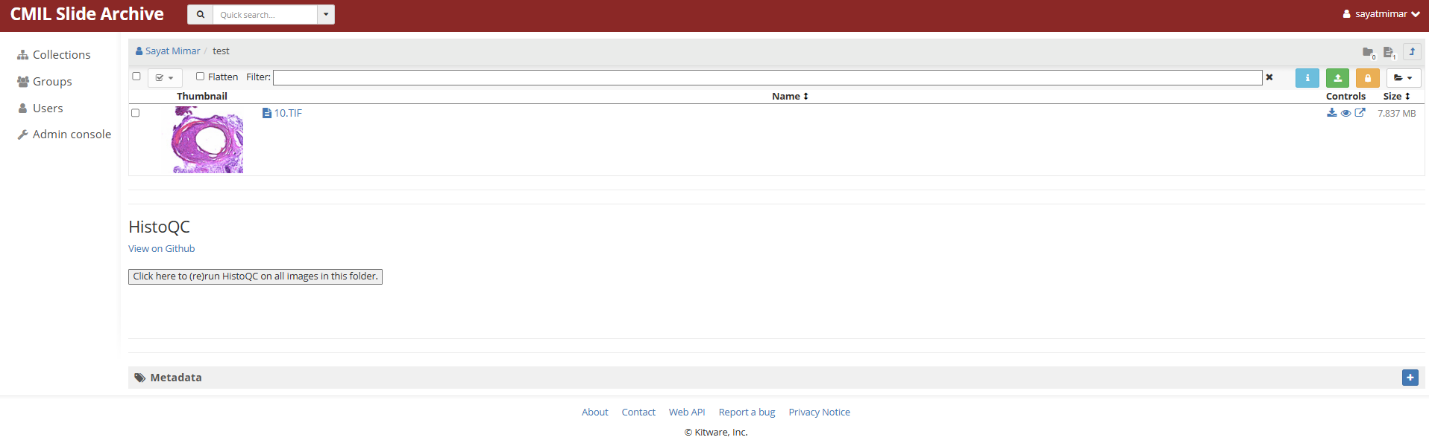


**Supp. Fig. 1 Navigating DSA** An image file can be uploaded through the green button on the top left corner of the page. Once uploaded, the image will appear in the folder.

**
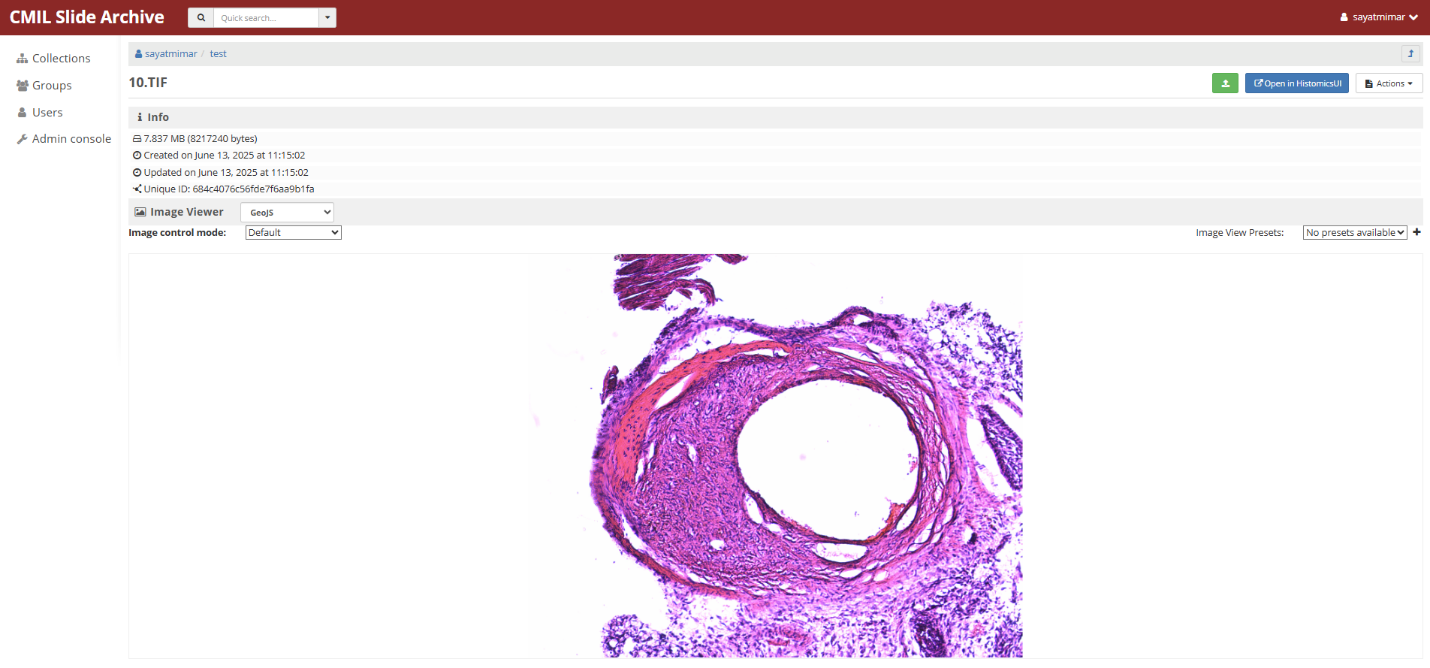
**

**Supp. Fig. 2**  **Navigating DSA (Cont.)** Upon clicking and opening a WSI in a separate page, a WSI can be viewed in HistomicUI (a plugin for visualizing large scale image data in cloud via DSA) by clicking ‘Open in HistomicsUI’ button.

For a selected image, the ingrown segmentation plugin can be run in the *Histomics* UI by clicking *Analyses/sarderlab/ingrownsegmentation/Segmentation*. After running the pipeline as described in the main text, users can visualize annotations in the UI (see **Supp. Fig. 3**).
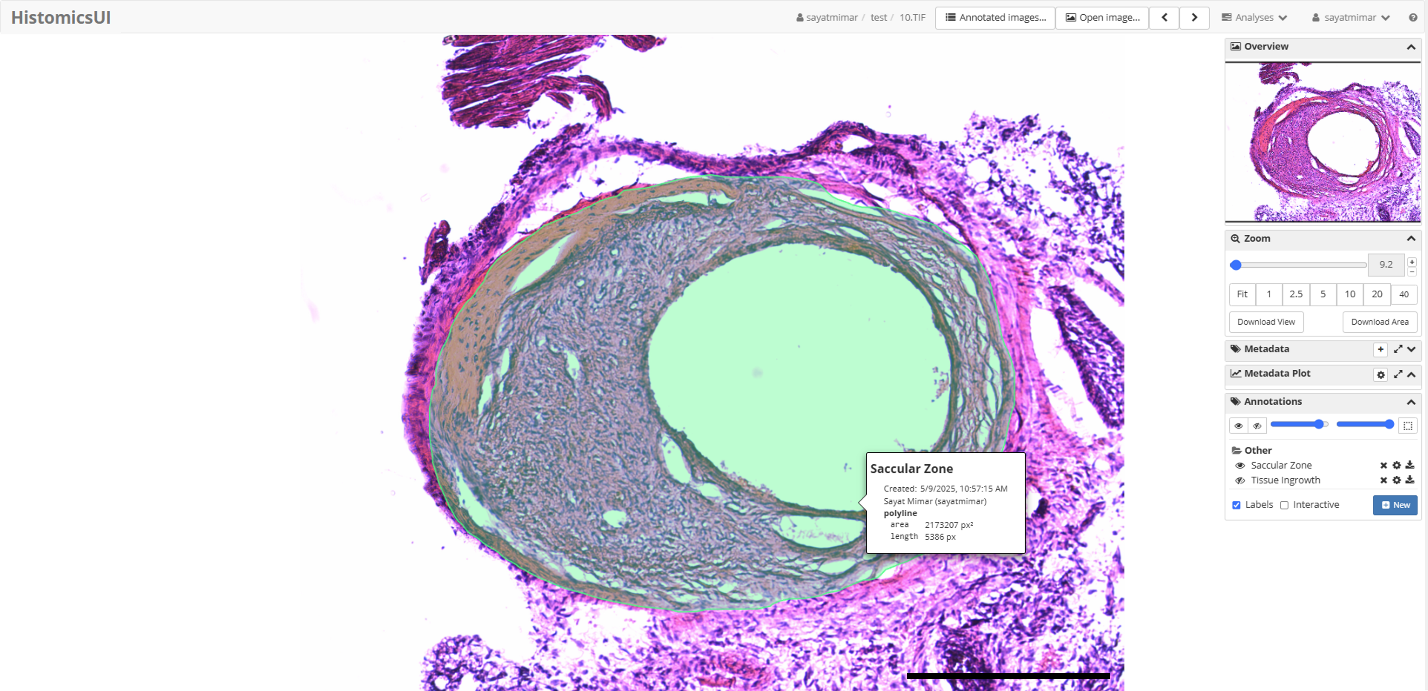

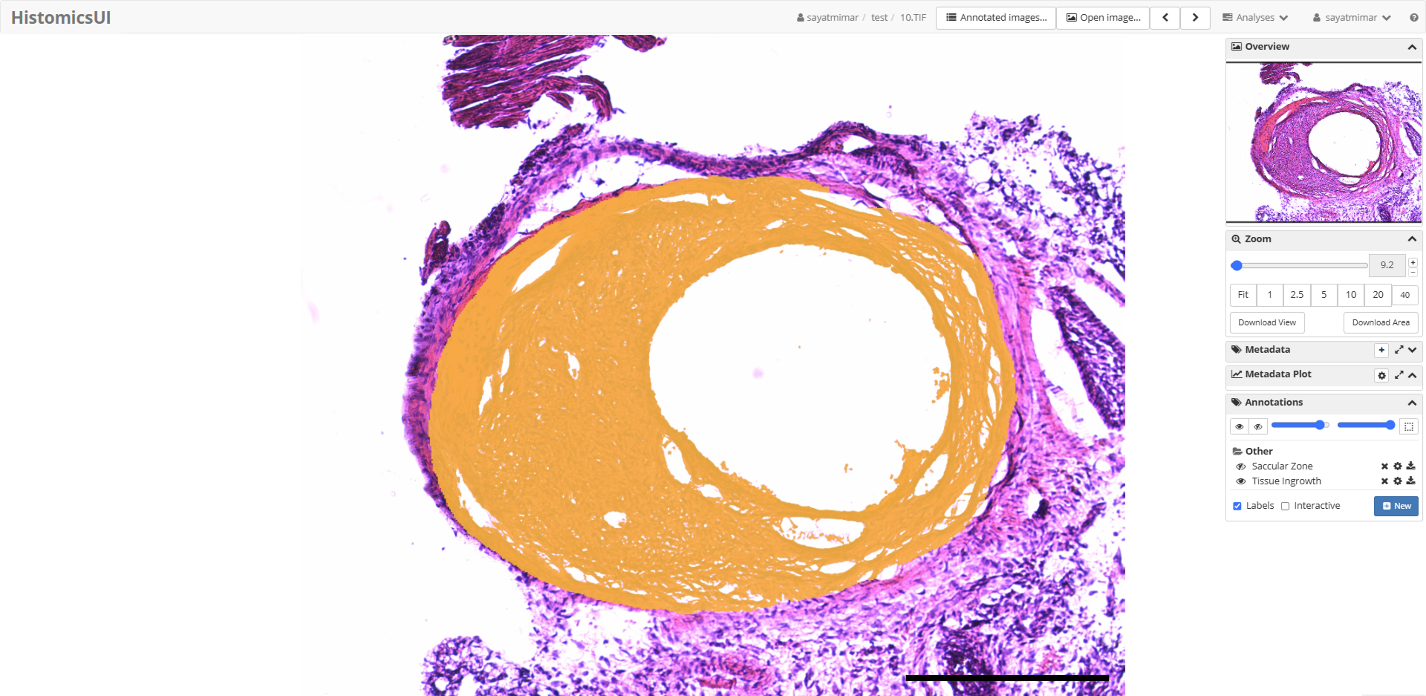


**B**

**A**

**Supp. Fig. 3** **Visualization of annotations in HistomicsUI** After running the Ingrown Segmentation plugin, the computational annotations (saccular zone in panel **A** and tissue ingrowth in panel **B**) can be found under the other section within Annotations and can be turned on/off by clicking the eye button on the left. After switching “Labels” field the UI displays morphometric measurements for the saccular zone. The scalebars correspond to 135 µm.
